# Electrophoretic deposition of ligand-free platinum nanoparticles on neural electrodes affects their impedance in vitro and in vivo with no negative effect on reactive gliosis

**DOI:** 10.1186/s12951-015-0154-9

**Published:** 2016-01-12

**Authors:** Svilen D. Angelov, Sven Koenen, Jurij Jakobi, Hans E. Heissler, Mesbah Alam, Kerstin Schwabe, Stephan Barcikowski, Joachim K. Krauss

**Affiliations:** Department of Neurosurgery, Hannover Medical School, Medical University Hannover, Carl-Neuberg-Str. 1, 30625 Hannover, Germany; Technical Chemistry I and Center for Nanointegration Duisburg-Essen (CENIDE), University of Duisburg-Essen, Universitätsstrasse 7, 45141 Essen, Germany

**Keywords:** In vitro, In vivo, Nanoparticles, Electrodes, Impedance, Electrophoretic deposition, Laser ablation, Deep brain stimulation, Neuronal recording, Biocompatibility

## Abstract

**Background:**

Electrodes for neural stimulation and recording are used for the treatment of neurological disorders. Their features critically depend on impedance and interaction with brain tissue. The effect of surface modification on electrode impedance was examined in vitro and in vivo after intracranial implantation in rats. Electrodes coated by electrophoretic deposition with platinum nanoparticles (NP; <10 and 50 nm) as well as uncoated references were implanted into the rat’s subthalamic nucleus. After postoperative recovery, rats were electrostimulated for 3 weeks. Impedance was measured before implantation, after recovery and then weekly during stimulation. Finally, local field potential was recorded and tissue-to-implant reaction was immunohistochemically studied.

**Results:**

Coating with NP significantly increased electrode’s impedance in vitro. Postoperatively, the impedance of all electrodes was temporarily further increased. This effect was lowest for the electrodes coated with particles <10 nm, which also showed the most stable impedance dynamics during stimulation for 3 weeks and the lowest total power of local field potential during neuronal activity recording. Histological analysis revealed that NP-coating did not affect glial reactions or neural cell-count.

**Conclusions:**

Coating with NP <10 nm may improve electrode’s impedance stability without affecting biocompatibility. Increased impedance after NP-coating may improve neural recording due to better signal-to-noise ratio.

## Background

Neuroprosthetic devices are used to modulate neuronal activity, e.g. during deep brain stimulation (DBS) for neuropsychiatric and movement disorders [1–4] or auditory brainstem implants [5, 6]. Additional recording of the neuronal activity is required for closed-loop (feedback) stimulation, e.g., for epilepsy control [7, 8]. The electrode quality is determined by numerous biological and physicochemical features, especially those that modify and/or stabilize impedance during chronic electrostimulation or recording, or those that influence tissue reaction [9]. For a smooth and stable metal surface, the current flow is limited by the maximum voltage that can be applied without causing tissue damage. With no Faradaic current, the metal interface will charge and the electrode should behave like a capacitor to prevent undesirable chemical reactions. One way to meet these requirements is to increase the electrode capacitance, while its geometric size is retained. This can be achieved by increasing the electrode surface area, e.g. by using nanostructured high surface area (HSA) materials. It has been shown that increased surface area can reduce the impedance and increase the charge transfer capability of electrodes [10]. In addition, nanoscale surface topography plays an important role in the interaction between the biological system and the implant [11–17]. The impedance and the current distribution also depend on the response of the neural tissue around the electrode contact, especially reactive gliosis and neuronal loss [18–23]. Future-generation implants are being designed with a great emphasis on reducing the tissue encapsulation problem and some of the most promising approaches include bioactive coatings and surface microstructuring [23]. In this context, electrophoretic deposition of nanoparticles (NP) from the same material derived by laser ablation in liquid is a suitable method to achieve homogenous coating without the use of chemical precursors and ligands [24–27].

**Fig. 1:**
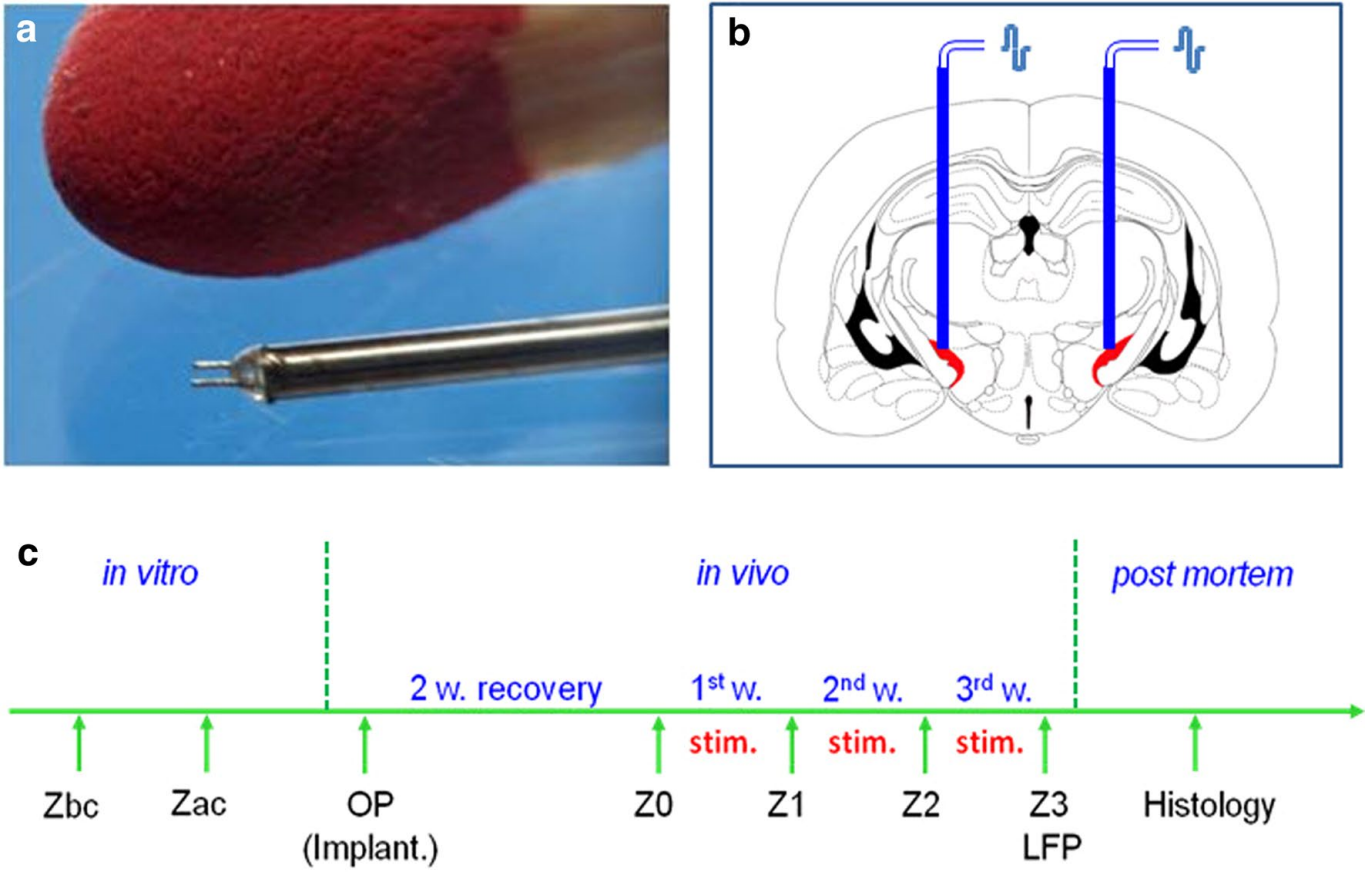
Picture of the electrode compared to a match stick (**a**) and schematic drawing of the implantation site in the subthalamic nucleus (**b**). Experimental design of the study (**c**). *Zbc and Zac* impedance measurements before and after coating; *OP* operation; *Z0–3* impedance measurements during stimulation; *stim.* electrostimulation; *LFP* recording of local field potential

Although electrophoretic deposition of NP has been applied before, e.g. to create a nanoroughness on stents [28], it has not been extensively investigated for neural electrodes in vivo. In this context, the impedance dynamics of coated platinum–iridium (Pt–Ir) stimulation electrodes was investigated in vitro and in vivo after intracranial implantation in a rat model. The corresponding electrodes were coated by electrophoretic deposition with NP of different sizes (<10, 50 nm, and a mixture of both) or left uncoated as controls. All NP were obtained by pulsed laser ablation in liquid in order to ensure that no ligands could interfere with the coating process and the biocompatibility of the electrode was not impaired by potentially toxic surfactants, required for synthesis [26]. Electrodes were implanted in the subthalamic nucleus (STN) of rats and after postoperative recovery the animals were electrostimulated for 3 weeks. Impedance was measured before implantation, after the recovery period and then weekly during stimulation (Fig. 1). Finally, neuronal oscillatory activity in the implantation site was recorded and local field potential (LFP) analyzed. Tissue-to-implant reaction was studied immunohistochemically after glial fibrillary acidic protein (GFAP)-staining and neuronal nuclear antigen (NeuN)-staining.

## Results

### Impedance in vitro (before and after coating)

**Fig. 2:**
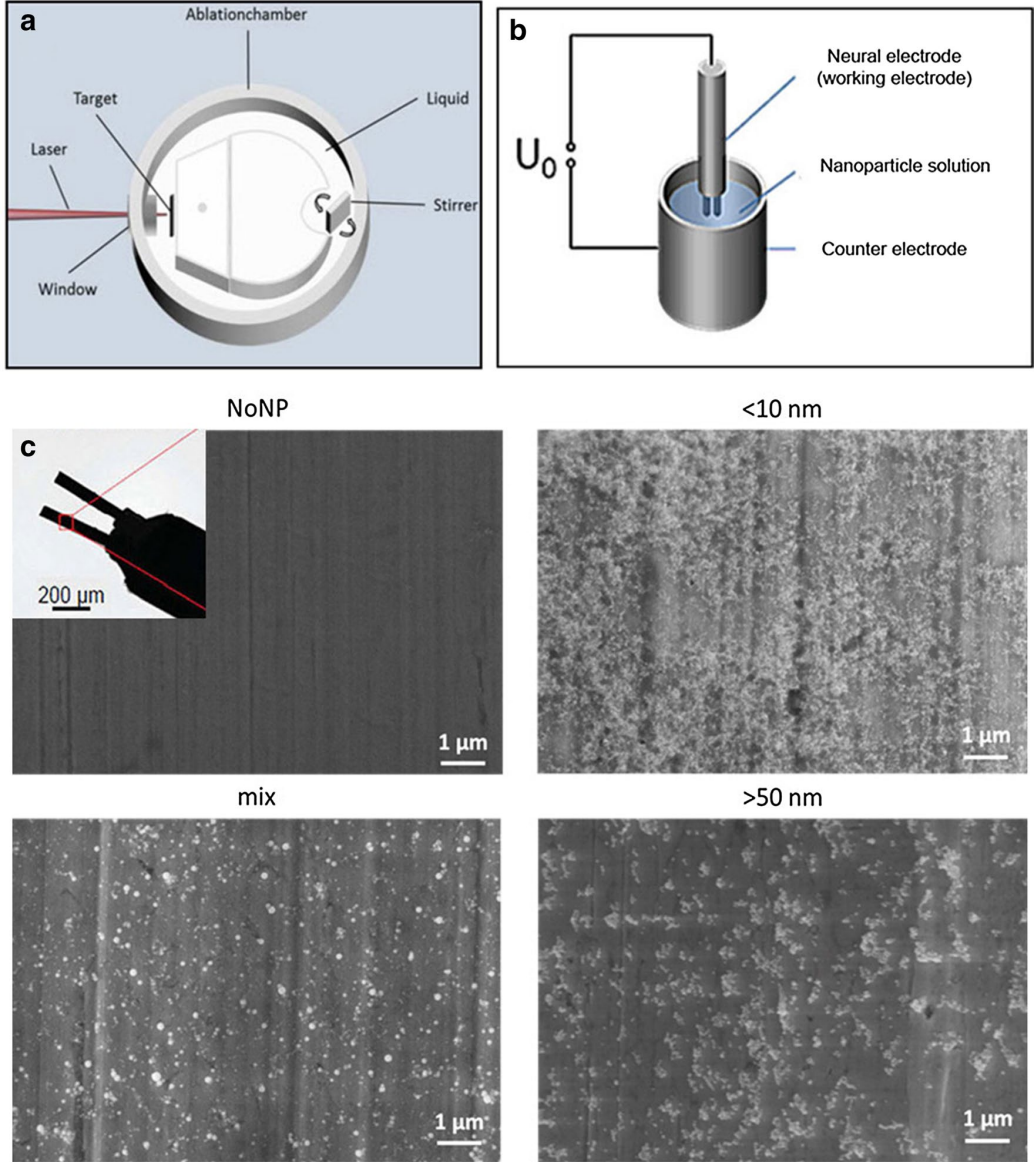
Schematic drawings of the **a** self-constructed laser ablation chamber to generate the nanoparticles, **b** electrophoretic deposition set-up to coat the electrode surface, and **c** exemplary SEM-images of the uncoated and coated contact surfaces (nanoparticles smaller than 10, 50 nm and mixture of both)

**Fig. 3:**
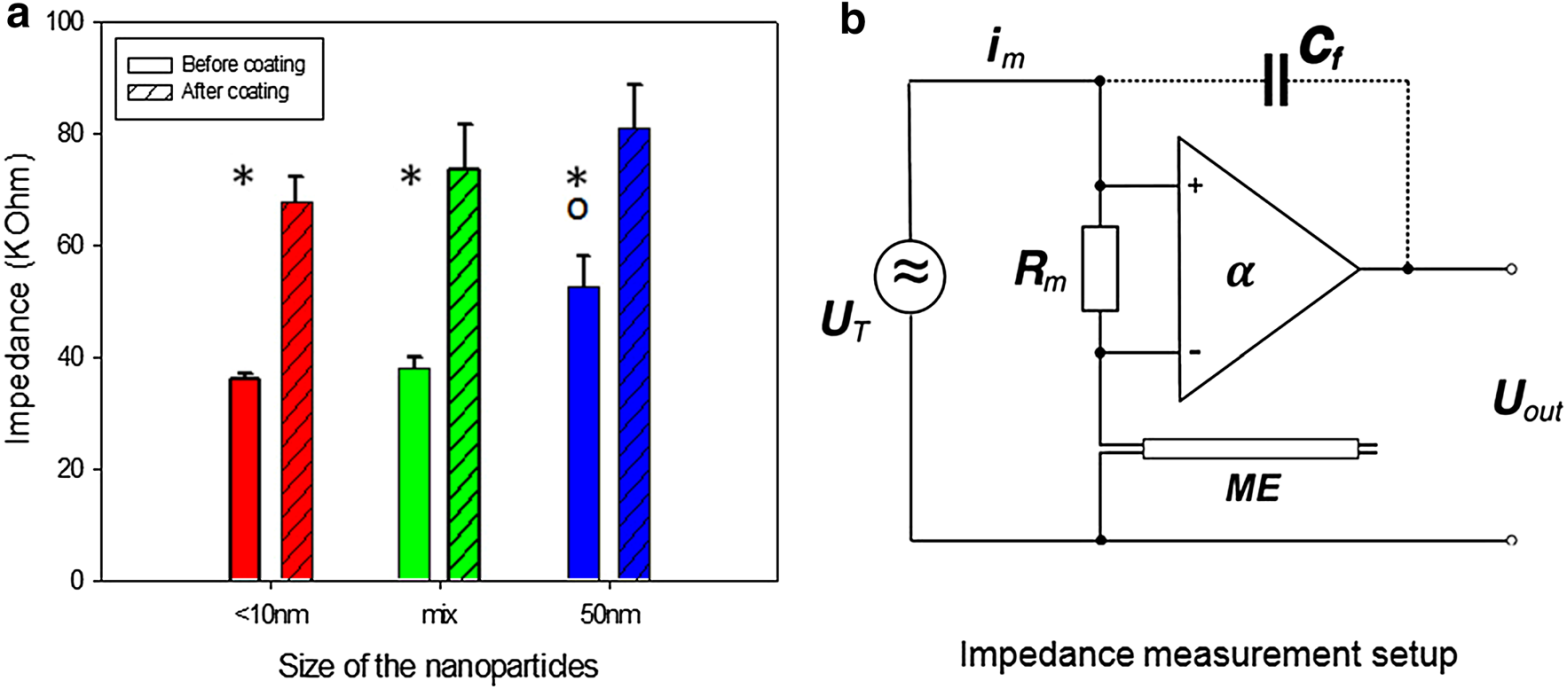
Effect of coatings with platinum nanoparticles <10, 50 nm and mixture of both (mix) on the impedance of stimulation electrodes in vitro (**a**). Data are means + SEM measured before and after coating. Significant differences within groups are indicated as *asterisks* (*), difference between groups is indicated as *circle* (o; ANOVA with post hoc Tukey’s test p < 0.05). Schematic diagram of the impedance measurement set-up (**b**). The sinusodial test voltage U_T_ drives the current i_m_ determined by the serial combination of R_m_ and the unknown microelectrode’s (ME) impedance. The voltage drop over R_m_ is amplified by α and registered as U_out._ Optionally, the feedback capacity C_f_ compensates for bandwidth narrowing. Applying mesh rules the impedance of ME can be eventually calculated

The preoperative (in vitro) evaluation of the coating effect includes measurements of the impedance in 93 electrodes, coated by electrophoretic deposition with ligand free platinum NP of three different sizes (<10 nm: n = 47; 50 nm: n = 22; mix: n = 24). Schematic drawings of the self-constructed laser ablation chamber and the electrophoretic deposition set-up, as well as exemplary SEM-images of the coated surface are shown in Fig. 2. Statistical evaluation with two-way analysis of variance (ANOVA) showed a significant effect for the factor particle size (F_2,185_ = 4.972, p = 0.009) and the factor test time (F_1,185_ = 60.960, p < 0.001), but no interaction between factors (F_2,185_ = 0.201, p < 0.819). Post-hoc analysis revealed that coating with NP of any size significantly increased impedances (all p values <0.001; Fig. 3).

In summary, these data clearly reveal that impedance is increased by any tested NP coating and this effect seems to be more pronounced with larger particles (50 nm) in comparison to smaller ones (10 nm).

### Impedance in vivo

**Fig. 4:**
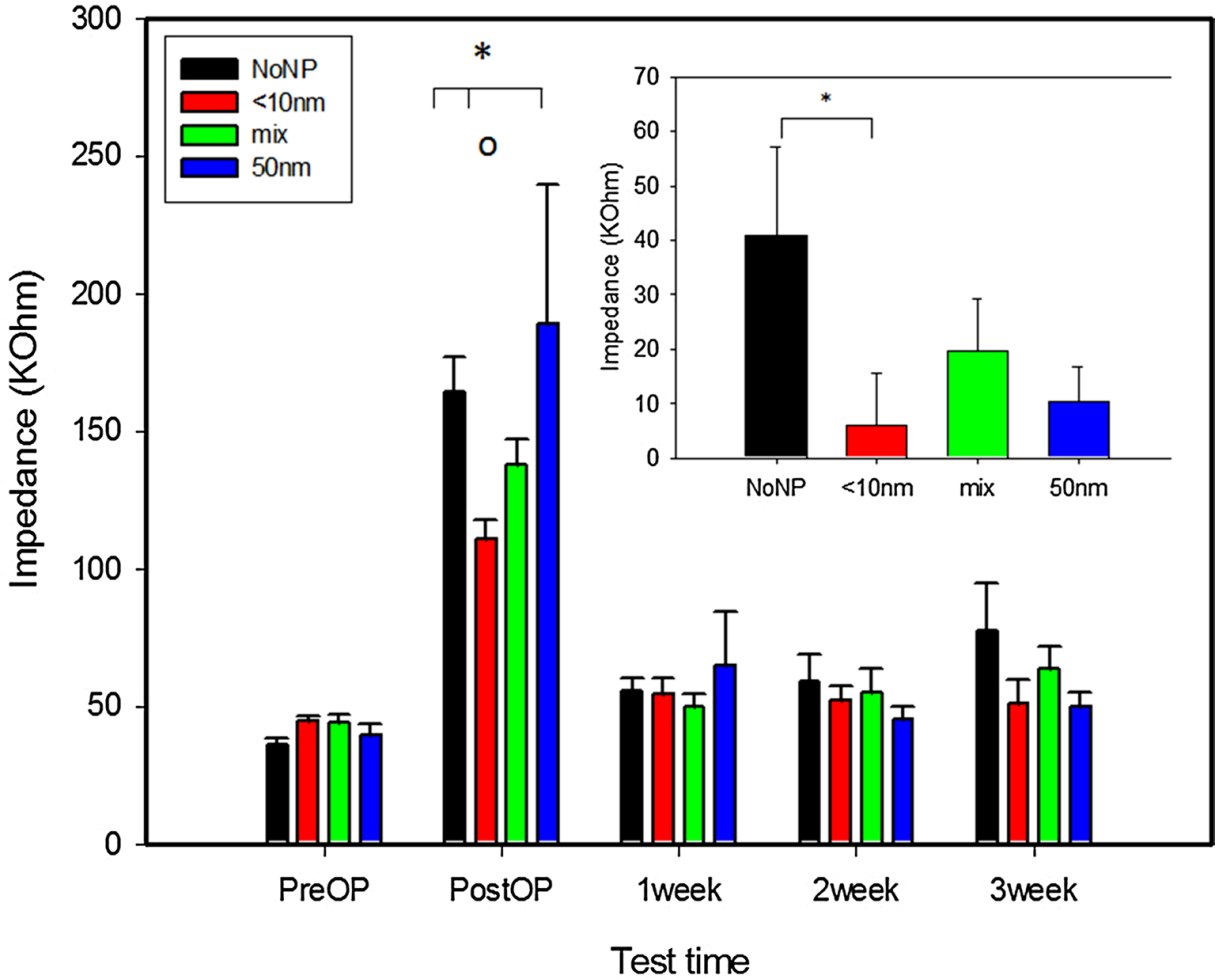
Impedance dynamics of electrodes coated with different nanoparticle sizes (<10 nm; 50 nm; mixture of both) and the uncoated group (NoNP) measured in vitro, 10 days postoperatively and after each of the three stimulation weeks (in vivo). Data are given as means + SEM. Significant difference between groups is indicated with (*asterisks*), while the *circle* (o) shows a significant effect between different test times (ANOVA with post hoc Tukey’s test p < 0.05). Inserted graph shows comparison of absolute impedance increase before operation and after the 3rd week of stimulation. Significant difference is indicated with* asterisk* (*; t test p < 0.05)

Four of the 31 bilaterally implanted animals lost the head socket during the stimulation period and the thread of two plugs got damaged. Additionally, 6 electrodes had no connection with the socket after surgery (broken wires/contacts) and one animal suffered severe tissue damage around the electrodes as a result of bleeding. Therefore 11 uncoated electrodes (in 6 rats), 11 electrodes (in 7 rats) coated with NP <10 nm, 10 electrodes (in 5 rats) coated with 50 nm NP and 10 electrodes (in 6 rats) coated with mix-sized NP were used for statistical evaluation of impedance in vivo (Fig. 4).

Statistical analysis with two-way ANOVA revealed an effect for the factor test time (F_4,209_ = 52.357, p < 0.001), no effect for the factor particles size (F_3,209_ = 0.826, p = 0.488), but an interaction between factors (F_12,209_ = 1.869, p = 0.042). Post hoc testing showed that impedance of both coated and uncoated electrodes was significantly higher on the first postoperative measurement than before operation and all following postoperative measurements (all p values <0.001). However, the first postoperative testing showed that impedance of the 10 nm coated electrodes was significantly lower than that of the 50 nm coated electrodes (p < 0.001) and the uncoated electrodes (p = 0.024). The impedances measured during the three stimulation weeks did not differ significantly between electrode groups, or between the different stimulation weeks within one group (Fig. 4). Nevertheless, comparison between the preoperative impedance and the one measured after the 3rd stimulation week showed a trend towards increased values for the uncoated group (p = 0.109), while for the coated electrodes almost no difference was found (all p values >0.800). Subsequent analysis of the absolute increase of impedance between pre-operative values and the impedance after 3 weeks of stimulation was significantly higher for the uncoated group as compared to the electrodes coated with 10 nm NP (*t* test, p = 0.042).

### Local field potential

Local field potential (LFP) was measured to further characterize the electrodes, since it is an electrophysiological signal generated by the current flowing from numerous neurons near the electrode tip, representing their input–output communications and may be used in the future as a signal for closed loop stimulation in patients with DBS electrodes.

**Fig. 5:**
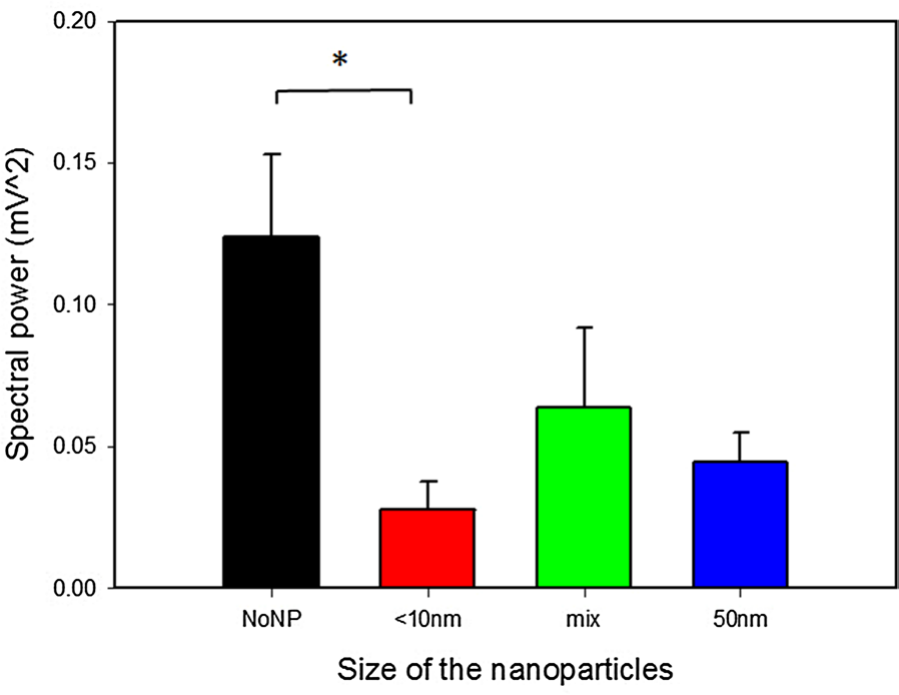
Total spectral power of the local field potential recorded in the subthalamic nucleus of the implanted animals. Data are means + SEM for the differently coated (nanoparticles smaller than 10 nm; 50 nm; mixture of both) and uncoated (NoNP) electrodes. Significant difference between groups is indicated with *asterisk* (*; ANOVA on Ranks, p < 0.05)

To guarantee the same source of neural activity, LFP was analyzed only from electrodes with the best STN localization i.e. 8 uncoated electrodes, 11 electrodes of the <10 nm group, 8 of the mix-sized group and 9 electrodes coated with 50 nm particles. The total spectral power for the <10 nm group was significantly lower compared to the value of the uncoated electrodes (p = 0.023; Fig. 5).

### Glial reaction and neuronal cell count (biocompatibility)

**Fig. 6:**
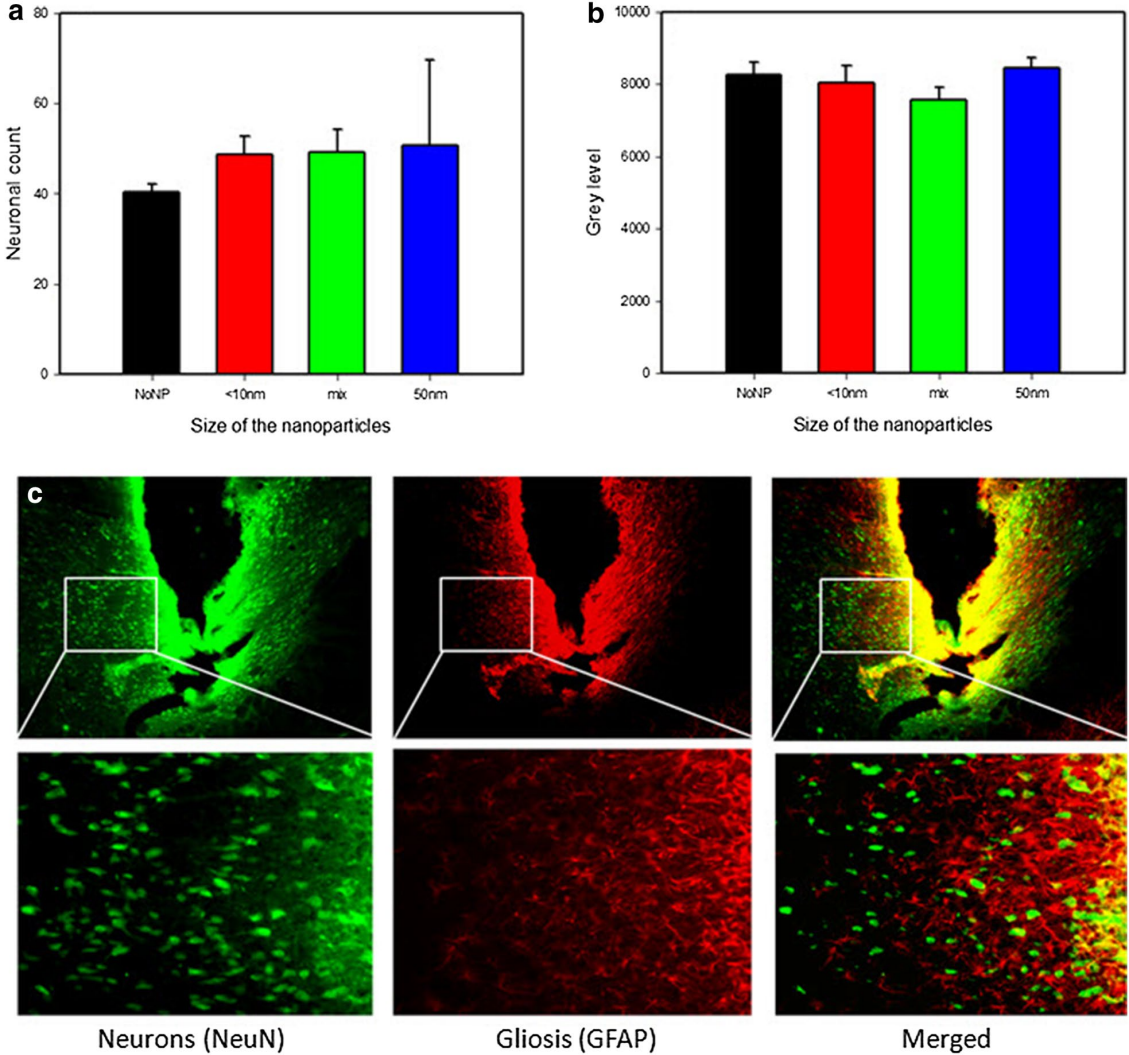
Neuronal count (**a**) and fluorescence intensity of the glial scar (**b**), measured around the electrode’s contact site 5 weeks after implantation for the different coated (nanoparticles smaller than 10 nm; 50 nm and mixture of both) electrodes and the uncoated (NoNP) group. Data are given as means + SEM. Photomicrographs (**c**) showing the neurons marked with NeuN (*green*) and the astrogliosis marked with GFAP *(red*) near the contact area of an electrode from the mix-sized group

For proper evaluation of gliosis and the number of neurons at the electrode tip, only sections through the midline of the wound canal (d ~ 75 µm) were used. This criterion was met by n = 6 sections from the uncoated group, n = 4 for the <10 nm group, n = 4 for 50 nm group and n = 7 for the electrodes coated with mixed size NP. Statistical evaluation with ANOVA of the densitometric data collected from the astrogliosis around the tip of the electrodes showed no significant difference between the groups (p = 0.338). The neuronal cell count did not differ significantly (p = 0.534; Fig. 6).

## Discussion

In the present in vitro study coating with NP led to an increase of the electrode impedance independent from the particle size. With regard to DBS, i.e., the chronic electrical stimulation of brain regions involved in the pathophysiology of certain neurological or neuropsychiatric disorders, such as Parkinson, dystonia and depression, electrodes with low impedance are preferred since this decreases power consumption. Nevertheless, high-impedance electrodes are needed for neural activity recording to improve the signal-to-noise ratio. With that regard, the increased impedance after coating is a worthwhile finding, because the quality of the recording directly depends on a good signal-to-noise ratio, which is primarily achieved with a high impedance of the recording electrode. Additionally, coating can increase electrode charge transfer capability, which is an important prerequisite for high-resolution stimulation or recording of neuronal activity. Enhanced spatial resolution can be achieved by reduction of the geometrical area of the microelectrodes. At the same time, absolute charge required for neuronal stimulation remains unchanged, thus posing the challenge to fabricate electrodes with extremely high effective surface area and consequently a high specific capacitance.

After implantation into the rat STN the impedance of all electrodes temporarily increased more than two fold independent from coating type, a phenomenon that has been repeatedly described before [29–31]. Notably, this effect was lowest in electrodes coated with <10 nm NP. Furthermore, these electrodes showed the most stable impedance during stimulation, while the impedance of uncoated electrodes increased over time. With regard to DBS, after initial determination of the stimulation intensity needed for symptom relief, correction of the amplitude is often required to obtain the same clinical effect, partially because the impedance changes with time. Even a moderate shift in electrode impedance may significantly change the volume of tissue affected by the stimulation and could alter the clinical effects [19, 32–34].

Electrodes with lower and/or more stable impedance would reduce both, the need of maintenance as well as power consumption. This impedance stability is also favorable for adaptive DBS, where feedback LFP signals are preferably recorded directly from the stimulating electrode to control when stimulation is delivered [35, 36].

The postoperatively enhanced impedance has been attributed to the tissue-to-implant response, which can be divided in acute and chronic phases [11, 23, 31]. The acute tissue reaction around the electrode tip starts with formation of a wound, followed by an inflammatory response, which leads to activation of microglia, astrocytes and macrophages. These acute physiological changes have been shown to appear as a spike in the impedance measurements [23, 30], followed by a decrease of impedance during the subsequent chronic reaction, which is characterized by moderate gliosis [9, 30] and roughly corresponds to the time of our electrostimulation period. After activation, the astrocytes begin to proliferate, forming a sheath around the implant culminating in a glial scar, which insulates the electrode from surrounding neurons and increases the impedance of the system. Gliosis extends the distance between the electrode and its target neurons, thus degrading the amplitude of the stimulation and recording signals [22, 23, 31, 37, 38].

Reactive astrocytes increase synthesis of glial fibrillary acidic protein (GFAP), which can be used to identify hypertrophic reactive astrocytes by immunocytochemical methods [39–42]. However, immunohistological analysis of the reactive astrogliosis around the electrode tip did not reveal differences between coated and uncoated electrodes. Labelling neurons with the neuronal nuclear antigen (NeuN), that can be used to detect neuronal cell loss around the implantation site possibly resulting from coating-induced neurotoxicity or electrostimulation, also did not reveal differences between electrodes. Our immunohistological findings presume that the coatings used on the electrode contact surface are rather safe for the host tissue. Important in this context is that laser ablation of metals in liquids generates NP without the use of chemical precursors and ligands [24–26, 43]. Since both the electrodes and the NP are made of the same highly biocompatible material (platinum with 10 % iridium), the absence of ligands and other chemical additives will reduce the time and efforts needed to test biocompatibility of coated electrodes, decreasing the number of the possibly confounding factors. Nevertheless, electrodes coated with <10 nm NP showed the lowest postoperative increase of impedance, which may indicate that coating the electrodes with NP of that size may, to some extent, improve their electrophysiological properties. Moreover, these electrodes showed the most stable impedance values between the different stimulation epochs, while the impedance of the uncoated group increased significantly over test time. Together, these findings may indicate that the acute and chronic tissue reaction (protein and cell adhesion, inflammation etc.) are less intensive in electrodes coated with <10 nm NP.

Recordings of neuronal activity have been also carried out with the nano-coated electrodes. The LFP is an electrophysiological signal obtained from nearby neuronal assemblies by low-pass filtering (usually cut off at 100–300 Hz) the extracellular electrical potential in the brain [44–46]. The LFP varies as a result of synaptic and postsynaptic activity and is believed to carry information about the synchronized neuronal input around the recording electrode and provides a tool to assess the input–output relations of neuronal activity [47]. Nevertheless, the origin of the LFP and how its recording quality depends on the electrode features are still poorly understood. Some recent studies show that electrode surface area and other electrode geometry factors can have a significant effect on LFP amplitude and spatial reach, while the effect of other variables, such as impedance could be of less importance for the LFP recording [36, 48].

Analyzing the LFP, we found that electrodes coated with NP <10 nm recorded LFP with significantly lower total power than the uncoated electrodes, while no changes were found in the relative spectral power of the different frequency bands. Since we did not measure significantly different impedance values or tissue reaction at the third stimulation week, it seems that the effect on LFP from the <10 nm coating is related to the surface geometry of the contacts. Indeed, with the NP <10 nm we achieved the most homogenous coating and it is also the one that increases the surface area the most. The exact mechanism of this surface-LFP interaction needs further investigation.

## Conclusions

Electrophoretic deposition of ligand-free platinum NP on the surface of three dimensional stimulation electrodes significantly affects their impedance in vivo and in vitro. Increased impedance could improve electrodes for neuronal activity recording, leading to a better signal to noise ratio, which is to be examined in the second phase of this project. Additionally, we showed that implant surface modification by deposition of <10 nm NP could improve impedance stability, which may have a positive clinical effect during longterm DBS. Finally, coatings with different size of the particles did not negatively affect the glial scar around the electrode and seem to be rather safe for the host tissue.

## Methods

### Animals

For this study 31 male Sprague–Dawley rats were used (Charles River Laboratories, Germany). The animals were kept in groups of 3–4 in standard Macrolon Type IV S cages (Techniplast, Hohenpeissenberg, Germany) under controlled ambient conditions (22 °C, 14 h light/10 h dark cycle, lights on at 07:00 a. m.). After surgery, each rat was kept in a standard Macrolon Type III cage. Rats received tap water ad libitum and 15 g rat-chow/animal/day.

The experimental protocols used in this study were in accordance with the national and international ethical guidelines, conducted in compliance with the German Animal Welfare Act and approved by the local authorities, which includes approval by an animal ethics committee (#AZ 14/1642).

### Experimental design

Platinum–iridium electrodes were electrophoretically coated with three different size groups of platinum NP (<10, 50 nm and mixture of both) or left uncoated for control. Impedance of the electrodes was measured in vitro before and after surface nanostructuring. Thereafter, the electrodes were bilaterally implanted in the STN of rats with NP coatings as follows: <10 nm—n = 9; 50 nm—n = 7; mixture—n = 8 and uncoated—n = 7. After 2 weeks of postoperative recovery the animals were stimulated for 3 weeks (Fig. 1). Impedance was assessed in vivo after the recovery period and after every stimulation week. Finally, all rats were sacrificed, perfused with paraformaldehyde and the brains were immunhistologically processed for GFAP- and NeuN-staining.

### Electrodes

Bipolar electrodes were made of two parallel Pt–Ir (90:10 %) wires insulated with Teflon (d = 0.0055″ with insulation and d = 0.003″ uninsulated; Science-Products GmbH, Hofheim, Germany), placed in a 0.55 × 17 mm stainless steel tube cut from a 24G syringe needle. At the contact end, both wires were uninsulated leaving a 500 µm long bare surface with about 250 µm intercontact distance. Plug pins were welded to the other end. The electrode tip was cleaned and conditioned before coating by immersing it in 65 % nitric acid for 15 min and then rinsing it thoroughly with distilled water. The first impedance measurement was done before coating, but after cleaning, to exclude changes induced by the cleaning procedure. Electrodes with impedance values between 20 and 65 KΩ were used for implantation.

### Laser ablation and electrophoretic deposition

The Pt NP used for this study were generated by pulsed laser ablation in liquid (PLAL)—a laser beam is focused on a bulk target of the desired material (platinum plate in this case) [49–51]. Ultrapure water was used as a solvent to guarantee a clean product, free from any unwanted impurities like organic ligands, salts or other chemicals. Ablation was conducted in a self-constructed ablation chamber (Fig. 2), which has been shown in more details by Nachev et al. [52]. A system containing a ns-pulsed Nd:YAG Laser (Rofin PowerLine E20) and a scanner with an F-Theta lens was chosen to generate the nanoparticles as described by Koenen et al. [53]. Afterwards, nanoparticles were centrifuged to isolate the ones of the desired size.

Electrophoretic deposition was used to deposit the nanoparticles on the electrode surface. This process requires a working and a counter electrode (Fig. 2), with the stimulation electrode, where the nanoparticles are deposited, serving as working electrode in this case [54]. Nanoparticles created by PLAL have a negative surface charges originating from partial surface oxidation and anion adsorption [55, 56]. This renders PLAL-generated particles ideally-suited for electrophoretic deposition, where an external electric field forces the nanoparticles on the electrode surface [53], affecting wettability (contact angle) of the implant metal surface [27].

### Impedance measurement

Electrode impedance (in vitro) before and after coating was calculated on the basis of Ohm’s law at a single frequency (200 Hz). The electrodes were immersed in 0.9 % NaCl and a sinusoidal test voltage (200 mV p–p) was applied to drive a current through the electrode and a serial measurement resistor (200 ± 1 % Ω). This current is proportional to the voltage drop across the measurement resistor, which was fed into a precision differential amplifier (AMP01, Analog Devices, Inc., Norwood, MA, USA). The amplifier output voltage allowed the calculation of the current amplitude and thus, by applying Ohm’s law, the estimation of electrode impedance by the ratio of electrode voltage and current. The existence of capacitive reactance was verified by phase shift of the test voltage and current. The same methodology was also used for postoperative (in vivo) impedance measurements. Additional details about the setup are shown in Fig. 3.

### Surgery

The rats were intraperitoneally anaesthetized with chloral hydrate (360 mg/kg) and fixed into a stereotaxic frame. Additionally, the surgical site was infiltrated with a local anesthetic (prilocainhydrochlorid 2 %). After incision and defining of bregma, two burr holes were drilled bilaterally above the target and two bipolar electrodes were implanted into the STN using the following coordinates (in mm) relative to bregma: anteroposterior: −3.8, mediolateral: ±2.5, dorsoventral: −8.0. The tooth bar was set to −3.3 mm. The electrodes and the socket were fixed to the skull with dental acrylic cement (Paladur^®^, Heraus Kulzer GmbH, Hanau, Germany). Four screws (1 × 2 mm) were wound to the skull as reinforcement. Antibiotics (marbofloxacin, 6.6 mg/kg) were applied for 8 days subcutaneously, starting 2 days preoperatively.

### Deep brain stimulation

After 2 weeks of postoperative recovery, continuous electrical stimulation was applied via a cable that was bite-protected by a metal spring-like shield. One side of the cable was connected to the socket on the skull, and the other to a stimulation device (Multichannel Systems STG2008, Software: Mc-Stimulus II). A swivel (Plastics one Inc, Roanoke, VA, USA) in the stimulation line allowed free movement of the rat without twisting the cable. For electrical stimulation symmetric, bipolar, rectangular pulses with duration of 160 µs were used. Frequency was 130 Hz and the pulse amplitude was experimentally determined (20 % below the individual motor reaction threshold). Stimulation parameters were controlled with an oscilloscope (Tektronix TDS2000C). During continuous stimulation, each rat was single housed in a standard Macrolon Type III cage. A 2 × 25 cm slot in the home cage lid allowed free movement of the animal with the cable attached.

### Local field potential

After the third week of stimulation, local field potential was recorded from the implanted Pt–Ir electrodes (Spike II software v.6). The signal was amplified 1000× using isolated amplifiers (Cambridge Electronic Design CED 1902), 0.5–100 Hz band-pass filtered and digitized (Cambridge Electronic Design CED 1401 Mark II) with a sampling rate of 1 kHz.

The discrete Fourier transform and its derivations, calculated according to the method of Halliday et al. [57], were used for total spectral power analysis. All data were visually inspected for artefacts and analyzed in MATLAB (The Mathworks, Natick, USA). Power spectra were calculated by dividing the waveform signal into a number of equal segments of 1.024 s (1024 data points without overlap) and each section was windowed (Hanning window). The magnitude of the discrete Fourier transform was squared and averaged to form the power spectrum of 1–100 Hz (delta to gamma waves), which has been most commonly used to analyze neural physiological processing, yielding a frequency resolution of 0.987 Hz.

### Histology

The implantation sites were histologically verified after the end of the experiments, using the atlas of Paxinos and Watson [58] as a reference. At the end of the 3 weeks stimulation period, i.e. 5 weeks after implantation, rats were deeply anaesthetized with an overdose of chloral hydrate and transcardially perfused with 4 % paraformaldehyde solution. The brains were removed from the cranial cavity, placed in 30 % sucrose/phosphate-buffered saline (PBS) solution for at least 12 h and cut on a freezing microtome (coronal plane) with a section width of 40 µm in three series. Nissl-staining, GFAP and NeuN immunofluorescent processing were used for localization and biocompatibility assessment. The slides were analyzed with a light microscope (Axio Imager Z1.m, Zeiss, Göttingen, Germany) and an imaging system (MetaMorph 7.1.3.0, Molecular Devices, CA, USA).

The first step of double immunohistochemical staining was incubating the sections for 10 min in 3 % hydrogen peroxide/10 % methanol/PBS (Biochrom GmbH, Berlin, Germany) to block endogenous peroxidase activity, followed by a 3 × 5 min washing procedure with PBS. Subsequently, non-specific sites were blocked at room temperature for 1 h in 5 % normal goat serum (Linaris GmbH, Dossenheim, Germany)/PBS. The next step was overnight incubation in the primary antibody solution—mouse anti-NeuN (Chemicon International, CA, USA) 1:1000 + rabbit anti-GFAP 1:1000 (Sigma-Aldrich, Germany) diluted in 1 % bovine serum albumin (BSA; Sigma-Aldrich, Germany)/0.3 % Triton X-100 (OmniChem, Louvain-la-Neuve, Belgium)/PBS. This step was followed by 3 × 5 min washing in PBS and then the sections were incubated (in the dark) at room temperature for 1 h in the secondary antibody solution—goat anti-Mouse Cy2 1:200 + goat anti-rabbit Cy3 1:200 (Dianova GmbH, Hamburg, Germany) in 1 % BSA/0.3 % Triton X-100/PBS. Next, the sections were again 3 × 5 min washed in PBS and transferred to a Petri dish with 0.7 % gelatin/distilled water solution for mounting on glass slides. After overnight drying at 4 °C, the slides were coverslipped using fluorescence mounting medium (Dako, Agilent Technologies, Denmark).

For the GFAP reactivity analysis and neuronal counting, monochrome pictures were taken with magnification of 100× and exposure time of 60 ms. The implantation site was oriented in the camera view field identically for all specimens. The pictures were thresholded subtracting the background. Quantitative densitometry of the gliosis was applied by measuring the intensity of fluorescence, always in a fixed-size rectangular test area aligned similarly to the edge of the electrode tip for every picture (adapted from Rothman et al. [59]).

### Statistical analysis

For statistical evaluation of the impedance dynamics in vitro and in vivo, data were analyzed by two way repeated measures ANOVA with time and particles size as factors, followed by post hoc Tukey’s test. One way ANOVA on ranks was used for statistical analysis of the glial scar area, neuronal cell count and total spectral power of the LFP. All tests were performed two-sided with p < 0.05 considered to be statistically significant.

## Abbreviations

ANOVA: analysis of variance
BSA: bovine serum albumin
DBS: deep brain stimulation
GFAP: glial fibrillary acidic protein
HSA: high surface area
LFP: local field potential
NeuN: neuronal nuclear antigen
NP: nanoparticles
PBS: phosphate-buffered saline
PLAL: pulsed laser ablation in liquid
Pt–Ir: platinum–iridium
STN: subthalamic nucleus
